# Transcriptional provirus silencing in the context of the integration site environment

**DOI:** 10.1186/1742-4690-10-S1-P83

**Published:** 2013-09-19

**Authors:** Filip Šenigl, Miroslav Auxt, Dalibor Miklík, Jiří Hejnar

**Affiliations:** 1 Department of Cellular and Viral Genetics, Institute of Molecular Genetics, Academy of Sciences of the Czech Republic, Prague, Czech Republic

## Background

Autonomous transcription of integrated retroviruses strongly depends on the genetic and epigenetic effects exerted by chromatin at the site of integration. These effects are mostly suppressive and proviral transcription can be ultimately silenced by mechanisms such as DNA methylation and histone modifications. Transcriptional silencing is a general feature of integrated retroviruses, however, the extent and frequency varies among the retroviral species. On that account, features determining the activity of individual integrated proviruses need to be identified.

## Materials and methods

To address the role of the integration site at the whole-genome-scale, we performed clonal analysis of provirus silencing with an ASLV-derived and a HIV-derived reporter vectors and correlated the transcriptional silencing with the epigenomic landscape of the respective integrations. The analysis was performed in the human K562 cell line and particularly in HCT116-derived DNMT-deficient cell lines in order to analyse the role of DNA methyltransferases in the provirus silencing.

## Results

We demonstrate efficient provirus silencing in human K562 and HCT116 cell lines, which is strongly but not absolutely dependent on the *de novo* DNA methyltransferase activity [1]. Proviruses integrated close to the transcription start sites of active genes into the regions enriched in H3K4 trimethylation (H3K4me3) display long-term stability of expression and are resistant to the transcriptional silencing even after overexpression of Dnmt3a or Dnmt3b. By contrast, proviruses in the intergenic regions tend to spontaneous transcriptional silencing even in *Dnmt3a-/- Dnmt3b-/-* cells. The silencing of proviruses within the transcribed regions of genes is accompanied by DNA methylation of long terminal repeats and dependent on the presence of *de novo* DNA methyltransferases. Converse, silencing in intergenic regions is DNA methylation-independent.

Since CpG islands were reported as DNA methylation-resistant genomic regions, we designed an ASLV-derived reporter vector modified by insertion of the CpG island core element [2] into its LTR promoter. Such LTR modification results in more variable integration site spectra of stably expressing proviruses.

## Conclusions

These findings indicate that the epigenomic features of integration sites are crucial for their permissiveness to the proviral expression. Both ASLV and HIV proviruses require similar epigenomic environment for their stable expression and the increased frequency of stable HIV proviruses is probably the result of the integration preference, which favours permissive genomic regions.
